# [μ-1,1′-Bis(diphenyl­phosphino)ferro­cene-κ^2^ *P*:*P*′]bis­[chloridogold(I)]–chloro­form–hexane (2/2/1)

**DOI:** 10.1107/S1600536810001418

**Published:** 2010-01-16

**Authors:** Nadine Meyer, Fabian Mohr, Edward R. T. Tiekink

**Affiliations:** a Fachbereich C – Anorganische Chemie, Bergische Universität Wuppertal, 42119 Wuppertal, Germany; b Department of Chemistry, University of Malaya, 50603 Kuala Lumpur, Malaysia

## Abstract

In the title mixed solvate, [Au_2_Fe(C_17_H_14_P)_2_Cl_2_]·CHCl_3_·0.5CH_3_(CH_2_)_4_CH_3_, the hexane solvent mol­ecule is disposed about an inversion centre. The Au atoms exist within nearly ideal linear coordination defined by *P*,*Cl*-donor sets, and when viewed down the P⋯P axis the Au atoms are *gauche* to each other. In the crystal structure, the chloro­form solvent mol­ecule is associated with the complex *via* a C—H⋯Cl contact, and the hexane solvent mol­ecules occupy voids defined by the remaining components of the structure.

## Related literature

For three polymorphs of the unsolvated title complex, see: Crespo *et al.* (2000 ▶); Constable *et al.* (2007 ▶); Segapelo *et al.* (2008 ▶). For solvated forms of the title complex, see: Hill *et al.* (1989 ▶); Canales *et al.* (1997 ▶). For a definition of a pseudo-polymorph, see: Nangia (2006 ▶). For background to related studies in gold chemistry, see: Gallenkamp *et al.* (2009 ▶).
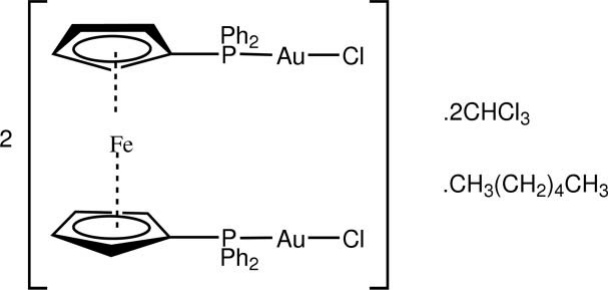


## Experimental

### 

#### Crystal data

[Au_2_Fe(C_17_H_14_P)_2_Cl_2_]·CHCl_3_·0.5C_6_H_14_
*M* *_r_* = 1181.64
Triclinic, 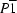

*a* = 11.631 (5) Å
*b* = 12.763 (5) Å
*c* = 14.530 (7) Å
α = 103.586 (7)°
β = 110.803 (9)°
γ = 92.802 (10)°
*V* = 1939.0 (15) Å^3^
*Z* = 2
Mo *K*α radiation
μ = 8.37 mm^−1^
*T* = 98 K
0.22 × 0.19 × 0.04 mm

#### Data collection

Rigaku AFC12/SATURN724 diffractometer
Absorption correction: multi-scan (*ABSCOR*; Higashi, 1995 ▶) *T*_min_ = 0.222, *T*_max_ = 1
19988 measured reflections
8010 independent reflections
7541 reflections with *I* > 2σ(*I*)
*R*_int_ = 0.041

#### Refinement

*R*[*F* ^2^ > 2σ(*F* ^2^)] = 0.039
*wR*(*F* ^2^) = 0.099
*S* = 1.08
8010 reflections
433 parameters
H-atom parameters constrained
Δρ_max_ = 2.53 e Å^−3^
Δρ_min_ = −2.36 e Å^−3^

### 

Data collection: *CrystalClear* (Rigaku/MSC, 2005 ▶); cell refinement: *CrystalClear*; data reduction: *CrystalClear*; program(s) used to solve structure: *SHELXS97* (Sheldrick, 2008 ▶); program(s) used to refine structure: *SHELXL97* (Sheldrick, 2008 ▶); molecular graphics: *ORTEP-3* (Farrugia, 1997 ▶) and *DIAMOND* (Brandenburg, 2006 ▶); software used to prepare material for publication: *publCIF* (Westrip, 2010 ▶).

## Supplementary Material

Crystal structure: contains datablocks global, I. DOI: 10.1107/S1600536810001418/hg2624sup1.cif

Structure factors: contains datablocks I. DOI: 10.1107/S1600536810001418/hg2624Isup2.hkl

Additional supplementary materials:  crystallographic information; 3D view; checkCIF report

## Figures and Tables

**Table:** 

Au1—Cl1	2.3131 (17)
Au1—P1	2.2413 (17)
Au2—Cl2	2.2988 (17)
Au2—P2	2.2357 (17)

**Table:** 

P1—Au1—Cl1	177.27 (5)
P2—Au2—Cl2	179.34 (5)

**Table 2:** Hydrogen-bond geometry (Å, °)

*D*—H⋯*A*	*D*—H	H⋯*A*	*D*⋯*A*	*D*—H⋯*A*
C35—H35⋯Cl2^i^	1.00	2.72	3.634 (8)	153

Symmetry code: (i) 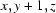.

**Fig. 1.**
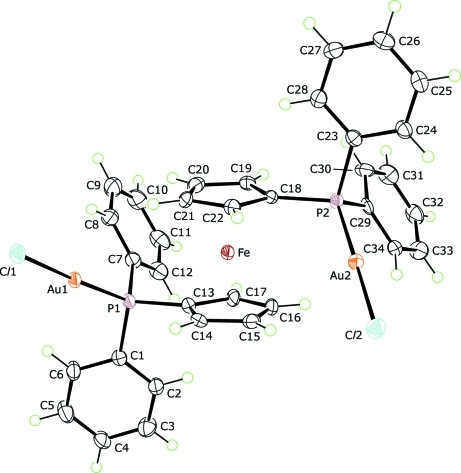
Molecular structure of the complex in (I), showing atom-labelling scheme and displacement ellipsoids at the 50% probability level.

**Fig. 2.**
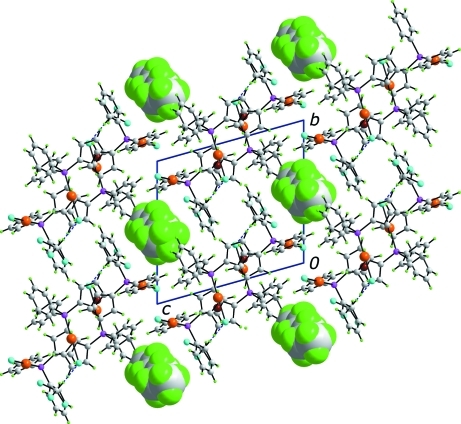
A view of the crystal packing in (I). The C–H···Cl interactions are shown as blue dashed lines, and the solvent hexane molecules are represented in space filling mode. Color code: Au, orange; Fe, brown; Cl, cyan; P, pink; C, grey; and H, green.

## supplementary crystallographic information

### Comment

In connection with on-going investigations of the biological activity of phosphinegold(I) thiolates (Gallenkamp *et al.*, 2009), the title mixed solvate, (I), was isolated and characterized crystallographically. The asymmetric unit of (I) comprises a dinuclear (C_34_H_28_FeP_2_)(AuCl)_2_ molecule, Fig. 1, a chloroform molecule, and half an hexane molecule which is located about an inversion centre. Each gold atom exists within a linear geometry defined by phosphorus and chloride atoms, Table 1. Each of the pairs of Au–Cl and Au–P bond distances are equal within 5σ, and the deviations from the ideal linear geometry are minimal, Table 1. The relative disposition of the P–Au–Cl chromophores is best described in terms of the Au1–P1···P1–Au2 torsion angle of 79.5 (6) ° and so may be regarded as being *gauche* (see discussion below). In the crystal structure, the chloroform molecule forms a C–H···Cl interaction with the Cl2 atom, Table 2. The hexane molecules occupy voids defined by the remaining components of the structure, Fig. 2.

Unsolvated polymorphic forms of the title complex have been characterized previously, *i.e*. in monoclinic space groups *P*2_1_/*n* (Crespo *et al.*, 2000) and *C*2/*c* (Segapelo *et al.*, 2008), and triclinic *P*1 (Constable *et al.*, 2007). In each of these polymorphs, the iron atom is located on a centre of inversion so that, from symmetry, the Au–P···P–Au torsion angle is 180 °. Two pseudo-polymorphs (Nangia, 2006) of the title complex are also known. In the 1:2 dichloromethane solvate, the iron atom is again located on a centre of inversion (Canales *et al.*, 1997). Finally, Hill *et al.* (1989) reported a chloroform solvate where the ratio of complex to chloroform was 3:2. One of the independent complex molecules was located about a centre of inversion. The second complex molecule adopted a *gauche* conformation with the Au–P···P–Au torsion angle being 117.78 (14) °.

### Experimental

Crystals of Au_2_Cl_2_(dppf) were adventitiously isolated by layering hexane onto a CDCl_3_ solution containing stoichiometric amounts of Au_2_Cl_2_(dppf) with 1,8-(Me_3_Sn_2_) naphthalene in an NMR tube.

### Refinement

The C-bound H atoms were geometrically placed (C–H = 0.95–1.00 Å) and refined as riding with *U_iso_*(H) = 1.2–1.5*U*_eq_(C). The maximum and minimum residual electron density peaks of 2.53 and -2.36 e Å^-3^, respectively, were located 1.50 Å and 0.84 Å from the Cl5 and Au2 atoms, respectively.

### Crystal data

**Table:**

[Au_2_Fe(C_17_H_14_P)_2_Cl_2_]·CHCl_3_·0.5C_6_H_14_	*Z* = 2
*M**_r_* = 1181.64	*F*(000) = 1126
Triclinic, *P*1	*D*_x_ = 2.024 Mg m^−^^3^
Hall symbol: -P 1	Mo *K*α radiation, λ = 0.71070 Å
*a* = 11.631 (5) Å	Cell parameters from 7032 reflections
*b* = 12.763 (5) Å	θ = 2.0–30.1°
*c* = 14.530 (7) Å	µ = 8.37 mm^−^^1^
α = 103.586 (7)°	*T* = 98 K
β = 110.803 (9)°	Block, orange
γ = 92.802 (10)°	0.22 × 0.19 × 0.04 mm
*V* = 1939.0 (15) Å^3^	

### Data collection

**Table:**

Rigaku AFC12K/SATURN724 diffractometer	8010 independent reflections
Radiation source: fine-focus sealed tube	7541 reflections with *I* > 2σ(*I*)
graphite	*R*_int_ = 0.041
ω scans	θ_max_ = 26.5°, θ_min_ = 1.9°
Absorption correction: multi-scan (*ABSCOR*; Higashi, 1995)	*h* = −14→14
*T*_min_ = 0.222, *T*_max_ = 1	*k* = −13→16
19988 measured reflections	*l* = −18→17

### Refinement

**Table:**

Refinement on *F*^2^	Primary atom site location: structure-invariant direct methods
Least-squares matrix: full	Secondary atom site location: difference Fourier map
*R*[*F*^2^ > 2σ(*F*^2^)] = 0.039	Hydrogen site location: inferred from neighbouring sites
*wR*(*F*^2^) = 0.099	H-atom parameters constrained
*S* = 1.08	*w* = 1/[σ^2^(*F*_o_^2^) + (0.0508*P*)^2^ + 6.3295*P*] where *P* = (*F*_o_^2^ + 2*F*_c_^2^)/3
8010 reflections	(Δ/σ)_max_ = 0.002
433 parameters	Δρ_max_ = 2.53 e Å^−^^3^
0 restraints	Δρ_min_ = −2.36 e Å^−^^3^

### Special details

**Table:**

Geometry. All e.s.d.'s (except the e.s.d. in the dihedral angle between two l.s. planes) are estimated using the full covariance matrix. The cell e.s.d.'s are taken into account individually in the estimation of e.s.d.'s in distances, angles and torsion angles; correlations between e.s.d.'s in cell parameters are only used when they are defined by crystal symmetry. An approximate (isotropic) treatment of cell e.s.d.'s is used for estimating e.s.d.'s involving l.s. planes.
Refinement. Refinement of *F*^2^ against ALL reflections. The weighted *R*-factor *wR* and goodness of fit *S* are based on *F*^2^, conventional *R*-factors *R* are based on *F*, with *F* set to zero for negative *F*^2^. The threshold expression of *F*^2^ > σ(*F*^2^) is used only for calculating *R*-factors(gt) *etc*. and is not relevant to the choice of reflections for refinement. *R*-factors based on *F*^2^ are statistically about twice as large as those based on *F*, and *R*- factors based on ALL data will be even larger.

### Fractional atomic coordinates and isotropic or equivalent isotropic displacement parameters (Å^2^)

**Table:**

	*x*	*y*	*z*	*U*_iso_*/*U*_eq_	
Au1	0.581716 (18)	0.161739 (17)	0.103915 (15)	0.01852 (8)	
Au2	0.735621 (18)	−0.075680 (16)	0.585129 (15)	0.01820 (8)	
Fe	0.74224 (7)	0.14693 (6)	0.41137 (6)	0.01708 (16)	
Cl1	0.37269 (12)	0.10792 (13)	0.00408 (10)	0.0270 (3)	
Cl2	0.73374 (14)	−0.26065 (11)	0.53407 (11)	0.0265 (3)	
Cl3	0.79521 (19)	0.55203 (16)	0.72594 (19)	0.0530 (5)	
Cl4	0.62630 (15)	0.67474 (14)	0.79754 (13)	0.0349 (4)	
Cl5	0.5292 (2)	0.47710 (15)	0.62931 (16)	0.0477 (4)	
P1	0.78213 (13)	0.21388 (11)	0.20723 (10)	0.0179 (3)	
P2	0.73597 (12)	0.10415 (11)	0.63297 (10)	0.0168 (3)	
C1	0.8941 (5)	0.1834 (4)	0.1473 (4)	0.0187 (11)	
C2	1.0085 (5)	0.1529 (5)	0.1977 (5)	0.0255 (12)	
H2	1.0277	0.1439	0.2644	0.031*	
C3	1.0948 (6)	0.1357 (6)	0.1512 (5)	0.0328 (14)	
H3	1.1730	0.1165	0.1867	0.039*	
C4	1.0667 (6)	0.1466 (6)	0.0532 (5)	0.0314 (14)	
H4	1.1240	0.1324	0.0203	0.038*	
C5	0.9536 (6)	0.1787 (6)	0.0035 (5)	0.0361 (15)	
H5	0.9349	0.1880	−0.0630	0.043*	
C6	0.8673 (6)	0.1974 (5)	0.0497 (4)	0.0283 (13)	
H6	0.7906	0.2195	0.0150	0.034*	
C7	0.8213 (6)	0.3588 (4)	0.2703 (4)	0.0222 (12)	
C8	0.7257 (6)	0.4229 (5)	0.2624 (5)	0.0263 (13)	
H8	0.6418	0.3902	0.2237	0.032*	
C9	0.7524 (7)	0.5335 (6)	0.3104 (5)	0.0355 (15)	
H9	0.6872	0.5764	0.3063	0.043*	
C10	0.8745 (7)	0.5806 (5)	0.3643 (5)	0.0360 (16)	
H10	0.8931	0.6569	0.3951	0.043*	
C11	0.9705 (7)	0.5189 (6)	0.3743 (5)	0.0354 (15)	
H11	1.0539	0.5529	0.4135	0.042*	
C12	0.9458 (6)	0.4076 (5)	0.3274 (5)	0.0286 (13)	
H12	1.0117	0.3651	0.3336	0.034*	
C13	0.8234 (5)	0.1463 (4)	0.3073 (4)	0.0191 (11)	
C14	0.7668 (5)	0.0387 (5)	0.2938 (4)	0.0216 (11)	
H14	0.7026	−0.0056	0.2337	0.026*	
C15	0.8246 (6)	0.0102 (5)	0.3874 (4)	0.0240 (12)	
H15	0.8053	−0.0569	0.4002	0.029*	
C16	0.9153 (5)	0.0984 (5)	0.4582 (4)	0.0267 (13)	
H16	0.9667	0.1010	0.5264	0.032*	
C17	0.9160 (5)	0.1821 (5)	0.4094 (4)	0.0226 (12)	
H17	0.9686	0.2503	0.4391	0.027*	
C18	0.6783 (5)	0.1609 (4)	0.5259 (4)	0.0167 (10)	
C19	0.7099 (5)	0.2673 (4)	0.5177 (4)	0.0204 (11)	
H19	0.7694	0.3245	0.5696	0.024*	
C20	0.6348 (5)	0.2711 (5)	0.4162 (4)	0.0229 (12)	
H20	0.6367	0.3319	0.3893	0.028*	
C21	0.5572 (5)	0.1694 (5)	0.3625 (4)	0.0232 (12)	
H21	0.4986	0.1506	0.2940	0.028*	
C22	0.5828 (5)	0.1011 (5)	0.4293 (4)	0.0193 (11)	
H22	0.5438	0.0285	0.4133	0.023*	
C23	0.6329 (5)	0.1383 (4)	0.7019 (4)	0.0188 (11)	
C24	0.6470 (5)	0.0969 (5)	0.7854 (4)	0.0223 (11)	
H24	0.7115	0.0546	0.8066	0.027*	
C25	0.5684 (6)	0.1168 (5)	0.8376 (4)	0.0261 (12)	
H25	0.5780	0.0873	0.8937	0.031*	
C26	0.4740 (6)	0.1807 (5)	0.8078 (5)	0.0261 (13)	
H26	0.4217	0.1970	0.8451	0.031*	
C27	0.4581 (6)	0.2194 (5)	0.7238 (5)	0.0273 (13)	
H27	0.3923	0.2603	0.7020	0.033*	
C28	0.5369 (5)	0.1997 (4)	0.6704 (4)	0.0209 (11)	
H28	0.5256	0.2276	0.6131	0.025*	
C29	0.8855 (5)	0.1835 (5)	0.7195 (4)	0.0191 (11)	
C30	0.8942 (6)	0.2915 (5)	0.7667 (5)	0.0253 (12)	
H30	0.8219	0.3261	0.7529	0.030*	
C31	1.0095 (6)	0.3513 (5)	0.8356 (5)	0.0306 (14)	
H31	1.0158	0.4266	0.8674	0.037*	
C32	1.1147 (6)	0.2991 (6)	0.8568 (5)	0.0318 (14)	
H32	1.1928	0.3393	0.9037	0.038*	
C33	1.1069 (5)	0.1903 (5)	0.8108 (5)	0.0280 (13)	
H33	1.1789	0.1553	0.8269	0.034*	
C34	0.9930 (5)	0.1316 (5)	0.7406 (4)	0.0209 (11)	
H34	0.9876	0.0570	0.7071	0.025*	
C35	0.6456 (7)	0.5917 (6)	0.6900 (6)	0.0385 (16)	
H35	0.6385	0.6357	0.6401	0.046*	
C36	0.5378 (6)	0.4984 (6)	0.9665 (5)	0.0350 (15)	
H36A	0.5662	0.5739	0.9693	0.042*	
H36B	0.4838	0.4610	0.8947	0.042*	
C37	0.6526 (7)	0.4386 (6)	0.9999 (5)	0.0357 (15)	
H37A	0.7074	0.4771	1.0711	0.043*	
H37B	0.6242	0.3638	0.9988	0.043*	
C38	0.7268 (7)	0.4324 (6)	0.9323 (6)	0.0436 (18)	
H38A	0.7500	0.3593	0.9179	0.065*	
H38B	0.8021	0.4864	0.9673	0.065*	
H38C	0.6763	0.4475	0.8680	0.065*	

### Atomic displacement parameters (Å^2^)

**Table:**

	*U*^11^	*U*^22^	*U*^33^	*U*^12^	*U*^13^	*U*^23^
Au1	0.01595 (12)	0.02468 (13)	0.01377 (12)	0.00351 (9)	0.00404 (9)	0.00541 (9)
Au2	0.01883 (12)	0.01858 (13)	0.01681 (12)	0.00345 (9)	0.00594 (9)	0.00520 (9)
Fe	0.0152 (4)	0.0221 (4)	0.0148 (4)	0.0046 (3)	0.0054 (3)	0.0068 (3)
Cl1	0.0172 (6)	0.0406 (8)	0.0188 (7)	0.0039 (6)	0.0032 (5)	0.0058 (6)
Cl2	0.0301 (7)	0.0217 (7)	0.0253 (7)	0.0048 (6)	0.0073 (6)	0.0066 (6)
Cl3	0.0501 (11)	0.0430 (10)	0.0916 (16)	0.0231 (9)	0.0479 (12)	0.0288 (10)
Cl4	0.0350 (9)	0.0389 (9)	0.0388 (9)	0.0144 (7)	0.0194 (7)	0.0147 (7)
Cl5	0.0600 (12)	0.0370 (9)	0.0527 (11)	0.0106 (8)	0.0284 (10)	0.0124 (8)
P1	0.0172 (7)	0.0220 (7)	0.0137 (6)	0.0022 (5)	0.0053 (5)	0.0044 (5)
P2	0.0155 (6)	0.0196 (7)	0.0145 (6)	0.0028 (5)	0.0044 (5)	0.0052 (5)
C1	0.018 (3)	0.017 (3)	0.018 (3)	−0.001 (2)	0.005 (2)	0.003 (2)
C2	0.024 (3)	0.034 (3)	0.021 (3)	0.008 (2)	0.009 (2)	0.010 (2)
C3	0.024 (3)	0.043 (4)	0.035 (4)	0.007 (3)	0.012 (3)	0.016 (3)
C4	0.029 (3)	0.048 (4)	0.023 (3)	0.011 (3)	0.015 (3)	0.011 (3)
C5	0.029 (3)	0.060 (5)	0.021 (3)	0.005 (3)	0.010 (3)	0.013 (3)
C6	0.022 (3)	0.045 (4)	0.019 (3)	0.008 (3)	0.006 (2)	0.013 (3)
C7	0.030 (3)	0.018 (3)	0.020 (3)	0.003 (2)	0.010 (2)	0.005 (2)
C8	0.027 (3)	0.024 (3)	0.024 (3)	0.005 (2)	0.007 (3)	0.003 (2)
C9	0.041 (4)	0.031 (3)	0.028 (3)	0.009 (3)	0.007 (3)	0.005 (3)
C10	0.052 (4)	0.022 (3)	0.031 (4)	0.001 (3)	0.016 (3)	0.002 (3)
C11	0.035 (4)	0.041 (4)	0.029 (3)	−0.004 (3)	0.016 (3)	0.003 (3)
C12	0.023 (3)	0.033 (3)	0.026 (3)	−0.002 (2)	0.009 (3)	0.002 (3)
C13	0.020 (3)	0.022 (3)	0.015 (3)	0.004 (2)	0.006 (2)	0.005 (2)
C14	0.025 (3)	0.025 (3)	0.017 (3)	0.008 (2)	0.012 (2)	0.004 (2)
C15	0.029 (3)	0.025 (3)	0.027 (3)	0.012 (2)	0.017 (3)	0.011 (2)
C16	0.022 (3)	0.046 (4)	0.017 (3)	0.017 (3)	0.008 (2)	0.014 (3)
C17	0.012 (3)	0.038 (3)	0.017 (3)	0.006 (2)	0.002 (2)	0.009 (2)
C18	0.013 (2)	0.024 (3)	0.012 (2)	0.006 (2)	0.004 (2)	0.004 (2)
C19	0.024 (3)	0.016 (3)	0.019 (3)	0.005 (2)	0.009 (2)	0.002 (2)
C20	0.022 (3)	0.024 (3)	0.028 (3)	0.008 (2)	0.012 (2)	0.011 (2)
C21	0.016 (3)	0.037 (3)	0.018 (3)	0.011 (2)	0.005 (2)	0.010 (2)
C22	0.015 (3)	0.021 (3)	0.018 (3)	0.000 (2)	0.004 (2)	0.001 (2)
C23	0.021 (3)	0.017 (3)	0.017 (3)	0.000 (2)	0.007 (2)	0.002 (2)
C24	0.022 (3)	0.027 (3)	0.016 (3)	0.002 (2)	0.006 (2)	0.004 (2)
C25	0.027 (3)	0.030 (3)	0.019 (3)	−0.001 (2)	0.007 (2)	0.007 (2)
C26	0.026 (3)	0.026 (3)	0.027 (3)	−0.001 (2)	0.014 (3)	0.002 (2)
C27	0.027 (3)	0.027 (3)	0.031 (3)	0.011 (2)	0.014 (3)	0.009 (3)
C28	0.022 (3)	0.018 (3)	0.019 (3)	0.001 (2)	0.004 (2)	0.003 (2)
C29	0.018 (3)	0.028 (3)	0.008 (2)	0.001 (2)	0.003 (2)	0.004 (2)
C30	0.022 (3)	0.027 (3)	0.023 (3)	0.006 (2)	0.010 (2)	−0.002 (2)
C31	0.034 (3)	0.023 (3)	0.026 (3)	−0.005 (3)	0.008 (3)	−0.003 (2)
C32	0.025 (3)	0.042 (4)	0.018 (3)	−0.001 (3)	0.002 (3)	0.001 (3)
C33	0.015 (3)	0.037 (4)	0.024 (3)	0.001 (2)	0.002 (2)	0.002 (3)
C34	0.022 (3)	0.025 (3)	0.015 (3)	0.006 (2)	0.006 (2)	0.004 (2)
C35	0.048 (4)	0.037 (4)	0.043 (4)	0.018 (3)	0.025 (4)	0.020 (3)
C36	0.033 (4)	0.043 (4)	0.027 (3)	0.002 (3)	0.011 (3)	0.005 (3)
C37	0.041 (4)	0.033 (4)	0.030 (3)	0.003 (3)	0.010 (3)	0.006 (3)
C38	0.046 (4)	0.038 (4)	0.046 (4)	0.009 (3)	0.020 (4)	0.006 (3)

### Geometric parameters (Å, °)

**Table:**

Au1—Cl1	2.3131 (17)	C14—H14	0.9500
Au1—P1	2.2413 (17)	C15—C16	1.417 (9)
Au2—Cl2	2.2988 (17)	C15—H15	0.9500
Au2—P2	2.2357 (17)	C16—C17	1.415 (8)
Fe—C18	2.026 (5)	C16—H16	0.9500
Fe—C22	2.039 (5)	C17—H17	0.9500
Fe—C14	2.045 (5)	C18—C19	1.432 (8)
Fe—C13	2.046 (5)	C18—C22	1.446 (7)
Fe—C15	2.049 (5)	C19—C20	1.437 (8)
Fe—C19	2.059 (5)	C19—H19	0.9500
Fe—C17	2.060 (6)	C20—C21	1.420 (8)
Fe—C16	2.060 (6)	C20—H20	0.9500
Fe—C20	2.069 (5)	C21—C22	1.417 (8)
Fe—C21	2.071 (5)	C21—H21	0.9500
Cl3—C35	1.767 (7)	C22—H22	0.9500
Cl4—C35	1.764 (7)	C23—C24	1.396 (8)
Cl5—C35	1.761 (8)	C23—C28	1.399 (8)
P1—C13	1.795 (6)	C24—C25	1.379 (8)
P1—C7	1.814 (6)	C24—H24	0.9500
P1—C1	1.817 (5)	C25—C26	1.404 (9)
P2—C18	1.798 (5)	C25—H25	0.9500
P2—C29	1.819 (6)	C26—C27	1.378 (9)
P2—C23	1.825 (5)	C26—H26	0.9500
C1—C6	1.396 (8)	C27—C28	1.393 (8)
C1—C2	1.397 (8)	C27—H27	0.9500
C2—C3	1.396 (8)	C28—H28	0.9500
C2—H2	0.9500	C29—C30	1.369 (8)
C3—C4	1.386 (9)	C29—C34	1.414 (8)
C3—H3	0.9500	C30—C31	1.404 (9)
C4—C5	1.392 (9)	C30—H30	0.9500
C4—H4	0.9500	C31—C32	1.394 (9)
C5—C6	1.394 (9)	C31—H31	0.9500
C5—H5	0.9500	C32—C33	1.376 (9)
C6—H6	0.9500	C32—H32	0.9500
C7—C8	1.401 (8)	C33—C34	1.393 (8)
C7—C12	1.410 (8)	C33—H33	0.9500
C8—C9	1.385 (9)	C34—H34	0.9500
C8—H8	0.9500	C35—H35	1.0000
C9—C10	1.377 (10)	C36—C36^i^	1.521 (13)
C9—H9	0.9500	C36—C37	1.554 (9)
C10—C11	1.383 (10)	C36—H36A	0.9900
C10—H10	0.9500	C36—H36B	0.9900
C11—C12	1.391 (9)	C37—C38	1.511 (10)
C11—H11	0.9500	C37—H37A	0.9900
C12—H12	0.9500	C37—H37B	0.9900
C13—C14	1.432 (8)	C38—H38A	0.9800
C13—C17	1.439 (8)	C38—H38B	0.9800
C14—C15	1.428 (8)	C38—H38C	0.9800
			
P1—Au1—Cl1	177.27 (5)	C16—C15—Fe	70.3 (3)
P2—Au2—Cl2	179.34 (5)	C14—C15—Fe	69.5 (3)
C18—Fe—C22	41.7 (2)	C16—C15—H15	125.6
C18—Fe—C14	144.2 (2)	C14—C15—H15	125.6
C22—Fe—C14	112.3 (2)	Fe—C15—H15	126.3
C18—Fe—C13	173.4 (2)	C17—C16—C15	108.0 (5)
C22—Fe—C13	144.7 (2)	C17—C16—Fe	69.9 (3)
C14—Fe—C13	41.0 (2)	C15—C16—Fe	69.4 (3)
C18—Fe—C15	113.2 (2)	C17—C16—H16	126.0
C22—Fe—C15	107.2 (2)	C15—C16—H16	126.0
C14—Fe—C15	40.8 (2)	Fe—C16—H16	126.3
C13—Fe—C15	68.5 (2)	C16—C17—C13	108.4 (5)
C18—Fe—C19	41.0 (2)	C16—C17—Fe	69.9 (3)
C22—Fe—C19	69.1 (2)	C13—C17—Fe	69.0 (3)
C14—Fe—C19	173.1 (2)	C16—C17—H17	125.8
C13—Fe—C19	134.4 (2)	C13—C17—H17	125.8
C15—Fe—C19	145.9 (2)	Fe—C17—H17	126.9
C18—Fe—C17	133.0 (2)	C19—C18—C22	107.6 (5)
C22—Fe—C17	171.6 (2)	C19—C18—P2	129.7 (4)
C14—Fe—C17	68.6 (2)	C22—C18—P2	122.6 (4)
C13—Fe—C17	41.0 (2)	C19—C18—Fe	70.7 (3)
C15—Fe—C17	67.8 (2)	C22—C18—Fe	69.6 (3)
C19—Fe—C17	111.0 (2)	P2—C18—Fe	126.4 (3)
C18—Fe—C16	108.3 (2)	C18—C19—C20	107.1 (5)
C22—Fe—C16	131.8 (2)	C18—C19—Fe	68.2 (3)
C14—Fe—C16	68.6 (2)	C20—C19—Fe	70.0 (3)
C13—Fe—C16	68.6 (2)	C18—C19—H19	126.5
C15—Fe—C16	40.3 (2)	C20—C19—H19	126.5
C19—Fe—C16	115.9 (2)	Fe—C19—H19	126.8
C17—Fe—C16	40.2 (2)	C21—C20—C19	109.1 (5)
C18—Fe—C20	68.6 (2)	C21—C20—Fe	70.0 (3)
C22—Fe—C20	67.8 (2)	C19—C20—Fe	69.3 (3)
C14—Fe—C20	132.9 (2)	C21—C20—H20	125.5
C13—Fe—C20	110.8 (2)	C19—C20—H20	125.5
C15—Fe—C20	171.0 (2)	Fe—C20—H20	126.9
C19—Fe—C20	40.7 (2)	C22—C21—C20	107.8 (5)
C17—Fe—C20	118.1 (2)	C22—C21—Fe	68.6 (3)
C16—Fe—C20	148.5 (3)	C20—C21—Fe	69.9 (3)
C18—Fe—C21	69.0 (2)	C22—C21—H21	126.1
C22—Fe—C21	40.3 (2)	C20—C21—H21	126.1
C14—Fe—C21	107.8 (2)	Fe—C21—H21	127.0
C13—Fe—C21	115.0 (2)	C21—C22—C18	108.4 (5)
C15—Fe—C21	131.4 (3)	C21—C22—Fe	71.0 (3)
C19—Fe—C21	68.6 (2)	C18—C22—Fe	68.7 (3)
C17—Fe—C21	148.0 (2)	C21—C22—H22	125.8
C16—Fe—C21	170.5 (3)	C18—C22—H22	125.8
C20—Fe—C21	40.1 (2)	Fe—C22—H22	126.0
C13—P1—C7	106.1 (3)	C24—C23—C28	119.5 (5)
C13—P1—C1	104.4 (3)	C24—C23—P2	118.4 (4)
C7—P1—C1	105.0 (3)	C28—C23—P2	122.1 (4)
C13—P1—Au1	110.77 (19)	C25—C24—C23	120.7 (5)
C7—P1—Au1	113.9 (2)	C25—C24—H24	119.7
C1—P1—Au1	115.77 (19)	C23—C24—H24	119.7
C18—P2—C29	107.8 (3)	C24—C25—C26	120.0 (6)
C18—P2—C23	104.4 (2)	C24—C25—H25	120.0
C29—P2—C23	104.2 (2)	C26—C25—H25	120.0
C18—P2—Au2	112.56 (18)	C27—C26—C25	119.4 (5)
C29—P2—Au2	115.27 (19)	C27—C26—H26	120.3
C23—P2—Au2	111.73 (18)	C25—C26—H26	120.3
C6—C1—C2	119.2 (5)	C26—C27—C28	121.1 (5)
C6—C1—P1	118.4 (4)	C26—C27—H27	119.5
C2—C1—P1	122.3 (4)	C28—C27—H27	119.5
C3—C2—C1	120.6 (6)	C27—C28—C23	119.4 (5)
C3—C2—H2	119.7	C27—C28—H28	120.3
C1—C2—H2	119.7	C23—C28—H28	120.3
C4—C3—C2	120.2 (6)	C30—C29—C34	119.9 (5)
C4—C3—H3	119.9	C30—C29—P2	120.8 (4)
C2—C3—H3	119.9	C34—C29—P2	119.3 (4)
C3—C4—C5	119.2 (6)	C29—C30—C31	120.4 (6)
C3—C4—H4	120.4	C29—C30—H30	119.8
C5—C4—H4	120.4	C31—C30—H30	119.8
C4—C5—C6	121.1 (6)	C32—C31—C30	119.3 (6)
C4—C5—H5	119.4	C32—C31—H31	120.3
C6—C5—H5	119.4	C30—C31—H31	120.3
C5—C6—C1	119.7 (6)	C33—C32—C31	120.9 (6)
C5—C6—H6	120.2	C33—C32—H32	119.6
C1—C6—H6	120.2	C31—C32—H32	119.6
C8—C7—C12	119.6 (5)	C32—C33—C34	119.8 (6)
C8—C7—P1	119.1 (5)	C32—C33—H33	120.1
C12—C7—P1	121.3 (5)	C34—C33—H33	120.1
C9—C8—C7	120.6 (6)	C33—C34—C29	119.8 (5)
C9—C8—H8	119.7	C33—C34—H34	120.1
C7—C8—H8	119.7	C29—C34—H34	120.1
C10—C9—C8	119.3 (6)	Cl5—C35—Cl4	111.4 (4)
C10—C9—H9	120.4	Cl5—C35—Cl3	111.0 (4)
C8—C9—H9	120.4	Cl4—C35—Cl3	110.2 (4)
C9—C10—C11	121.3 (6)	Cl5—C35—H35	108.1
C9—C10—H10	119.4	Cl4—C35—H35	108.1
C11—C10—H10	119.4	Cl3—C35—H35	108.1
C10—C11—C12	120.4 (6)	C36^i^—C36—C37	112.6 (7)
C10—C11—H11	119.8	C36^i^—C36—H36A	109.1
C12—C11—H11	119.8	C37—C36—H36A	109.1
C11—C12—C7	118.8 (6)	C36^i^—C36—H36B	109.1
C11—C12—H12	120.6	C37—C36—H36B	109.1
C7—C12—H12	120.6	H36A—C36—H36B	107.8
C14—C13—C17	107.5 (5)	C38—C37—C36	113.2 (6)
C14—C13—P1	122.8 (4)	C38—C37—H37A	108.9
C17—C13—P1	129.7 (4)	C36—C37—H37A	108.9
C14—C13—Fe	69.5 (3)	C38—C37—H37B	108.9
C17—C13—Fe	70.0 (3)	C36—C37—H37B	108.9
P1—C13—Fe	127.4 (3)	H37A—C37—H37B	107.8
C15—C14—C13	107.4 (5)	C37—C38—H38A	109.5
C15—C14—Fe	69.7 (3)	C37—C38—H38B	109.5
C13—C14—Fe	69.5 (3)	H38A—C38—H38B	109.5
C15—C14—H14	126.3	C37—C38—H38C	109.5
C13—C14—H14	126.3	H38A—C38—H38C	109.5
Fe—C14—H14	126.0	H38B—C38—H38C	109.5
C16—C15—C14	108.8 (5)		
			
Cl1—Au1—P1—C13	38.5 (11)	C20—Fe—C17—C13	−90.0 (4)
Cl1—Au1—P1—C7	−81.1 (11)	C21—Fe—C17—C13	−51.5 (6)
Cl1—Au1—P1—C1	157.1 (11)	C29—P2—C18—C19	−21.3 (5)
Cl2—Au2—P2—C18	−16 (5)	C23—P2—C18—C19	89.1 (5)
Cl2—Au2—P2—C29	−141 (4)	Au2—P2—C18—C19	−149.5 (4)
Cl2—Au2—P2—C23	101 (4)	C29—P2—C18—C22	160.9 (4)
C13—P1—C1—C6	162.1 (5)	C23—P2—C18—C22	−88.7 (4)
C7—P1—C1—C6	−86.4 (5)	Au2—P2—C18—C22	32.6 (5)
Au1—P1—C1—C6	40.1 (5)	C29—P2—C18—Fe	73.3 (4)
C13—P1—C1—C2	−21.8 (5)	C23—P2—C18—Fe	−176.4 (3)
C7—P1—C1—C2	89.7 (5)	Au2—P2—C18—Fe	−55.0 (4)
Au1—P1—C1—C2	−143.8 (4)	C22—Fe—C18—C19	−118.2 (4)
C6—C1—C2—C3	−0.5 (9)	C14—Fe—C18—C19	−172.7 (4)
P1—C1—C2—C3	−176.6 (5)	C13—Fe—C18—C19	47.8 (19)
C1—C2—C3—C4	−1.3 (10)	C15—Fe—C18—C19	151.6 (3)
C2—C3—C4—C5	2.3 (11)	C17—Fe—C18—C19	71.1 (4)
C3—C4—C5—C6	−1.6 (11)	C16—Fe—C18—C19	108.7 (4)
C4—C5—C6—C1	−0.2 (11)	C20—Fe—C18—C19	−37.9 (3)
C2—C1—C6—C5	1.3 (9)	C21—Fe—C18—C19	−81.1 (3)
P1—C1—C6—C5	177.5 (5)	C14—Fe—C18—C22	−54.5 (5)
C13—P1—C7—C8	−113.5 (5)	C13—Fe—C18—C22	166.0 (18)
C1—P1—C7—C8	136.3 (5)	C15—Fe—C18—C22	−90.2 (4)
Au1—P1—C7—C8	8.6 (5)	C19—Fe—C18—C22	118.2 (4)
C13—P1—C7—C12	66.1 (5)	C17—Fe—C18—C22	−170.8 (3)
C1—P1—C7—C12	−44.1 (5)	C16—Fe—C18—C22	−133.1 (3)
Au1—P1—C7—C12	−171.7 (4)	C20—Fe—C18—C22	80.3 (3)
C12—C7—C8—C9	−0.3 (9)	C21—Fe—C18—C22	37.1 (3)
P1—C7—C8—C9	179.4 (5)	C22—Fe—C18—P2	116.2 (5)
C7—C8—C9—C10	1.6 (10)	C14—Fe—C18—P2	61.6 (5)
C8—C9—C10—C11	−2.3 (10)	C13—Fe—C18—P2	−77.8 (19)
C9—C10—C11—C12	1.9 (11)	C15—Fe—C18—P2	26.0 (4)
C10—C11—C12—C7	−0.6 (10)	C19—Fe—C18—P2	−125.6 (5)
C8—C7—C12—C11	−0.2 (9)	C17—Fe—C18—P2	−54.6 (5)
P1—C7—C12—C11	−179.9 (5)	C16—Fe—C18—P2	−17.0 (4)
C7—P1—C13—C14	155.6 (4)	C20—Fe—C18—P2	−163.5 (4)
C1—P1—C13—C14	−93.8 (5)	C21—Fe—C18—P2	153.3 (4)
Au1—P1—C13—C14	31.5 (5)	C22—C18—C19—C20	−0.6 (6)
C7—P1—C13—C17	−26.9 (6)	P2—C18—C19—C20	−178.7 (4)
C1—P1—C13—C17	83.7 (5)	Fe—C18—C19—C20	59.5 (4)
Au1—P1—C13—C17	−151.1 (4)	C22—C18—C19—Fe	−60.1 (3)
C7—P1—C13—Fe	67.4 (4)	P2—C18—C19—Fe	121.8 (4)
C1—P1—C13—Fe	178.1 (3)	C22—Fe—C19—C18	38.9 (3)
Au1—P1—C13—Fe	−56.7 (4)	C14—Fe—C19—C18	141.9 (19)
C18—Fe—C13—C14	144.5 (18)	C13—Fe—C19—C18	−173.2 (3)
C22—Fe—C13—C14	−51.6 (5)	C15—Fe—C19—C18	−51.1 (5)
C15—Fe—C13—C14	38.1 (3)	C17—Fe—C19—C18	−132.2 (3)
C19—Fe—C13—C14	−172.6 (3)	C16—Fe—C19—C18	−88.5 (4)
C17—Fe—C13—C14	118.5 (5)	C20—Fe—C19—C18	118.8 (5)
C16—Fe—C13—C14	81.6 (4)	C21—Fe—C19—C18	82.3 (3)
C20—Fe—C13—C14	−132.2 (3)	C18—Fe—C19—C20	−118.8 (5)
C21—Fe—C13—C14	−88.8 (4)	C22—Fe—C19—C20	−79.9 (4)
C18—Fe—C13—C17	26 (2)	C14—Fe—C19—C20	23 (2)
C22—Fe—C13—C17	−170.1 (4)	C13—Fe—C19—C20	68.0 (4)
C14—Fe—C13—C17	−118.5 (5)	C15—Fe—C19—C20	−169.9 (4)
C15—Fe—C13—C17	−80.4 (4)	C17—Fe—C19—C20	109.0 (4)
C19—Fe—C13—C17	68.9 (4)	C16—Fe—C19—C20	152.7 (3)
C16—Fe—C13—C17	−36.9 (4)	C21—Fe—C19—C20	−36.5 (3)
C20—Fe—C13—C17	109.3 (4)	C18—C19—C20—C21	0.4 (6)
C21—Fe—C13—C17	152.8 (3)	Fe—C19—C20—C21	58.8 (4)
C18—Fe—C13—P1	−99.3 (18)	C18—C19—C20—Fe	−58.4 (4)
C22—Fe—C13—P1	64.6 (6)	C18—Fe—C20—C21	−82.5 (3)
C14—Fe—C13—P1	116.2 (5)	C22—Fe—C20—C21	−37.4 (3)
C15—Fe—C13—P1	154.3 (4)	C14—Fe—C20—C21	63.0 (4)
C19—Fe—C13—P1	−56.4 (5)	C13—Fe—C20—C21	104.5 (3)
C17—Fe—C13—P1	−125.3 (5)	C15—Fe—C20—C21	20.6 (16)
C16—Fe—C13—P1	−162.2 (4)	C19—Fe—C20—C21	−120.7 (5)
C20—Fe—C13—P1	−16.0 (4)	C17—Fe—C20—C21	149.1 (3)
C21—Fe—C13—P1	27.4 (5)	C16—Fe—C20—C21	−172.8 (4)
C17—C13—C14—C15	0.3 (6)	C18—Fe—C20—C19	38.2 (3)
P1—C13—C14—C15	178.2 (4)	C22—Fe—C20—C19	83.2 (4)
Fe—C13—C14—C15	−59.7 (4)	C14—Fe—C20—C19	−176.3 (3)
C17—C13—C14—Fe	60.0 (4)	C13—Fe—C20—C19	−134.8 (3)
P1—C13—C14—Fe	−122.1 (4)	C15—Fe—C20—C19	141.2 (14)
C18—Fe—C14—C15	−55.0 (5)	C17—Fe—C20—C19	−90.2 (4)
C22—Fe—C14—C15	−90.8 (4)	C16—Fe—C20—C19	−52.1 (6)
C13—Fe—C14—C15	118.5 (5)	C21—Fe—C20—C19	120.7 (5)
C19—Fe—C14—C15	168.8 (18)	C19—C20—C21—C22	0.0 (6)
C17—Fe—C14—C15	80.3 (4)	Fe—C20—C21—C22	58.3 (4)
C16—Fe—C14—C15	37.0 (4)	C19—C20—C21—Fe	−58.3 (4)
C20—Fe—C14—C15	−170.7 (3)	C18—Fe—C21—C22	−38.3 (3)
C21—Fe—C14—C15	−133.6 (4)	C14—Fe—C21—C22	103.8 (4)
C18—Fe—C14—C13	−173.5 (3)	C13—Fe—C21—C22	147.3 (3)
C22—Fe—C14—C13	150.7 (3)	C15—Fe—C21—C22	64.7 (4)
C15—Fe—C14—C13	−118.5 (5)	C19—Fe—C21—C22	−82.5 (3)
C19—Fe—C14—C13	50 (2)	C17—Fe—C21—C22	−178.2 (4)
C17—Fe—C14—C13	−38.3 (3)	C16—Fe—C21—C22	37.2 (15)
C16—Fe—C14—C13	−81.5 (4)	C20—Fe—C21—C22	−119.5 (5)
C20—Fe—C14—C13	70.8 (4)	C18—Fe—C21—C20	81.2 (3)
C21—Fe—C14—C13	107.9 (3)	C22—Fe—C21—C20	119.5 (5)
C13—C14—C15—C16	0.2 (6)	C14—Fe—C21—C20	−136.7 (3)
Fe—C14—C15—C16	−59.4 (4)	C13—Fe—C21—C20	−93.1 (4)
C13—C14—C15—Fe	59.6 (4)	C15—Fe—C21—C20	−175.8 (3)
C18—Fe—C15—C16	−91.3 (4)	C19—Fe—C21—C20	37.1 (3)
C22—Fe—C15—C16	−135.5 (3)	C17—Fe—C21—C20	−58.6 (6)
C14—Fe—C15—C16	120.1 (5)	C16—Fe—C21—C20	156.8 (12)
C13—Fe—C15—C16	81.8 (4)	C20—C21—C22—C18	−0.4 (6)
C19—Fe—C15—C16	−57.6 (5)	Fe—C21—C22—C18	58.7 (4)
C17—Fe—C15—C16	37.4 (3)	C20—C21—C22—Fe	−59.1 (4)
C20—Fe—C15—C16	169.2 (13)	C19—C18—C22—C21	0.6 (6)
C21—Fe—C15—C16	−173.2 (3)	P2—C18—C22—C21	178.9 (4)
C18—Fe—C15—C14	148.6 (3)	Fe—C18—C22—C21	−60.2 (4)
C22—Fe—C15—C14	104.5 (4)	C19—C18—C22—Fe	60.8 (3)
C13—Fe—C15—C14	−38.3 (3)	P2—C18—C22—Fe	−120.9 (4)
C19—Fe—C15—C14	−177.6 (4)	C18—Fe—C22—C21	119.5 (5)
C17—Fe—C15—C14	−82.6 (4)	C14—Fe—C22—C21	−91.6 (4)
C16—Fe—C15—C14	−120.1 (5)	C13—Fe—C22—C21	−57.8 (5)
C20—Fe—C15—C14	49.2 (16)	C15—Fe—C22—C21	−134.8 (3)
C21—Fe—C15—C14	66.7 (4)	C19—Fe—C22—C21	81.2 (4)
C14—C15—C16—C17	−0.6 (6)	C17—Fe—C22—C21	173.4 (15)
Fe—C15—C16—C17	−59.5 (4)	C16—Fe—C22—C21	−172.3 (3)
C14—C15—C16—Fe	58.9 (4)	C20—Fe—C22—C21	37.3 (3)
C18—Fe—C16—C17	−136.2 (3)	C14—Fe—C22—C18	149.0 (3)
C22—Fe—C16—C17	−176.8 (3)	C13—Fe—C22—C18	−177.3 (4)
C14—Fe—C16—C17	81.8 (4)	C15—Fe—C22—C18	105.8 (3)
C13—Fe—C16—C17	37.6 (3)	C19—Fe—C22—C18	−38.3 (3)
C15—Fe—C16—C17	119.2 (5)	C17—Fe—C22—C18	53.9 (17)
C19—Fe—C16—C17	−92.5 (4)	C16—Fe—C22—C18	68.3 (4)
C20—Fe—C16—C17	−57.6 (6)	C20—Fe—C22—C18	−82.2 (3)
C21—Fe—C16—C17	151.5 (12)	C21—Fe—C22—C18	−119.5 (5)
C18—Fe—C16—C15	104.6 (3)	C18—P2—C23—C24	174.6 (4)
C22—Fe—C16—C15	64.0 (4)	C29—P2—C23—C24	−72.5 (5)
C14—Fe—C16—C15	−37.4 (3)	Au2—P2—C23—C24	52.6 (5)
C13—Fe—C16—C15	−81.6 (3)	C18—P2—C23—C28	−2.1 (5)
C19—Fe—C16—C15	148.3 (3)	C29—P2—C23—C28	110.9 (5)
C17—Fe—C16—C15	−119.2 (5)	Au2—P2—C23—C28	−124.0 (4)
C20—Fe—C16—C15	−176.8 (4)	C28—C23—C24—C25	−0.7 (8)
C21—Fe—C16—C15	32.3 (15)	P2—C23—C24—C25	−177.4 (4)
C15—C16—C17—C13	0.7 (6)	C23—C24—C25—C26	−1.0 (9)
Fe—C16—C17—C13	−58.4 (4)	C24—C25—C26—C27	2.5 (9)
C15—C16—C17—Fe	59.2 (4)	C25—C26—C27—C28	−2.4 (9)
C14—C13—C17—C16	−0.6 (6)	C26—C27—C28—C23	0.8 (9)
P1—C13—C17—C16	−178.4 (4)	C24—C23—C28—C27	0.8 (8)
Fe—C13—C17—C16	59.0 (4)	P2—C23—C28—C27	177.4 (4)
C14—C13—C17—Fe	−59.6 (4)	C18—P2—C29—C30	63.6 (5)
P1—C13—C17—Fe	122.6 (5)	C23—P2—C29—C30	−46.9 (5)
C18—Fe—C17—C16	64.0 (5)	Au2—P2—C29—C30	−169.7 (4)
C22—Fe—C17—C16	16.7 (18)	C18—P2—C29—C34	−118.7 (4)
C14—Fe—C17—C16	−81.7 (4)	C23—P2—C29—C34	130.8 (4)
C13—Fe—C17—C16	−120.0 (5)	Au2—P2—C29—C34	8.0 (5)
C15—Fe—C17—C16	−37.6 (4)	C34—C29—C30—C31	0.5 (9)
C19—Fe—C17—C16	105.6 (4)	P2—C29—C30—C31	178.2 (5)
C20—Fe—C17—C16	150.0 (4)	C29—C30—C31—C32	−1.2 (9)
C21—Fe—C17—C16	−171.4 (4)	C30—C31—C32—C33	0.3 (10)
C18—Fe—C17—C13	−176.1 (3)	C31—C32—C33—C34	1.2 (10)
C22—Fe—C17—C13	136.6 (15)	C32—C33—C34—C29	−1.9 (9)
C14—Fe—C17—C13	38.2 (3)	C30—C29—C34—C33	1.1 (8)
C15—Fe—C17—C13	82.4 (4)	P2—C29—C34—C33	−176.6 (4)
C19—Fe—C17—C13	−134.4 (3)	C36^i^—C36—C37—C38	178.6 (7)
C16—Fe—C17—C13	120.0 (5)		

Symmetry codes: (i) −*x*+1, −*y*+1, −*z*+2.

### Hydrogen-bond geometry (Å, °)

**Table:**

*D*—H···*A*	*D*—H	H···*A*	*D*···*A*	*D*—H···*A*
C35—H35···Cl2^ii^	1.00	2.72	3.634 (8)	153

Symmetry codes: (ii) *x*, *y*+1, *z*.
